# Ecosystem experiment reveals benefits of natural and simulated beaver dams to a threatened population of steelhead (*Oncorhynchus mykiss*)

**DOI:** 10.1038/srep28581

**Published:** 2016-07-04

**Authors:** Nicolaas Bouwes, Nicholas Weber, Chris E. Jordan, W. Carl Saunders, Ian A. Tattam, Carol Volk, Joseph M. Wheaton, Michael M. Pollock

**Affiliations:** 1 Eco Logical Research, Inc., PO BOX 706, Providence, Utah, 84332, USA; 2 Watershed Sciences Department, Utah State University, 5210 Old Main Hill, Logan, Utah 84322, USA; 3 Northwest Fisheries Science Center, 2725 Montlake Blvd E., Seattle, Washington 98112, USA; 4 Oregon Department of Fish and Wildlife, Eastern Oregon University, 203 Badgley Hall, One University Boulevard, LaGrande, Oregon 97850, USA; 5 South Fork Research, Inc. 44842 SE 145th Street, North Bend, Washington, 98045, USA

## Abstract

Beaver have been referred to as ecosystem engineers because of the large impacts their dam building activities have on the landscape; however, the benefits they may provide to fluvial fish species has been debated. We conducted a watershed-scale experiment to test how increasing beaver dam and colony persistence in a highly degraded incised stream affects the freshwater production of steelhead (*Oncorhynchus mykiss*). Following the installation of beaver dam analogs (BDAs), we observed significant increases in the density, survival, and production of juvenile steelhead without impacting upstream and downstream migrations. The steelhead response occurred as the quantity and complexity of their habitat increased. This study is the first large-scale experiment to quantify the benefits of beavers and BDAs to a fish population and its habitat. Beaver mediated restoration may be a viable and efficient strategy to recover ecosystem function of previously incised streams and to increase the production of imperiled fish populations.

Beaver in Eurasia and North America were once abundant and ubiquitous^1^. Their dense and barbed fur has great felting properties, and as early as the 1500s, intense trapping to provide pelts mainly for making hats occurred throughout Eurasia^2^. By the early 1700s, beaver were nearly extirpated in Eurasia, and North America became the new source of pelts for international commerce. The exploration, settlement, and many territorial claims of North America by several European countries were driven mainly by the search for beaver-trapping opportunities^2^.

When Lewis and Clark explored the Pacific Northwest in 1805, salmon and steelhead coexisted with beavers in very high densities^1^,^3^. Fur trade in this region began around 1810, attracting pioneers to settle the area. When the British and United States jointly occupied the Oregon Territories (which included the Columbia River Basin), the Hudson Bay Company implemented their “scorched earth” or “fur desert” policy to eliminate all fur-bearing animals, in an attempt to discourage American settlement^2^,^4^. As a result, beaver were nearly extirpated from the region by 1900. Around this time, a decrease in the great harvests of Pacific salmon and steelhead was first perceived. Anadromous salmon and steelhead populations have since declined precipitously in the Columbia River Basin, leading to their listing under the U.S. Endangered Species Act (ESA)^5^,^6^. Agriculture, timber harvest, mining, grazing, urban development, and water storage and hydroelectric dam construction are commonly cited as the causes for salmonid habitat degradation and population declines^7^, with rare mention of the loss of beaver and their ability to alter aquatic ecosystems with their dam-building activities^8^.

Human activities, including the removal of beaver, have exacerbated the occurrence of stream channel incision, where a rapid down-cutting of the stream bed disconnects the channel from its floodplain^8^,^9^. Channel incision is a ubiquitous environmental problem in the Columbia River Basin and throughout the world^10^,^11^,^12^. Consequences of channel incision include a lowering of the water table, decreased base flows, warmer water temperatures, and reduced morphological complexity leading to a substantial loss of riparian plant biomass and diversity, and declines in fish populations and other aquatic organisms^13^. The succession of channel incision can be described by four phases: phase 1) rapid incision and disconnection of the floodplain, phase 2) widening of the incised trench, phase 3) building of inset floodplains and long-term aggradation, and phase 4) returning to a channel in dynamic equilibrium that is reconnected to its floodplain^13^. Incised channels can take centuries to millennia to fully recover to the dynamic equilibrium phase^14^. We hypothesized that beaver dams or simulated beaver dams that we construct (referred to as beaver dam analogs or BDAs) can greatly accelerate the incision recovery process^14^. We further hypothesized that advancing channel incision recovery would alter the hydrologic, thermal, geomorphic, and vegetation characteristics of stream reaches and their associated riparian habitats, which in turn would improve habitat conditions for steelhead (Fig. 1).

Ecosystem scale experiments have greatly improved our understanding of watershed processes and are a powerful method for evaluating and predicting responses to environmental change^15^. Such experiments generally involve large-scale perturbations simulating human impacts (e.g., logging, nutrient additions) and have led to changes in strategies to minimize environmental degradation^16^,^17^,^18^. While insightful, these experiments are often costly and destructive, and do not necessarily address mechanisms of recovery processes. Implementing restoration as a watershed-scale experiment could greatly increase our understanding of ecosystem function, and our ability to achieve recovery goals while making better and more efficient use of the financial investments in mitigation^19^. We describe the results of a watershed-scale experiment designed to test whether constructing beaver dam analogs to encourage natural beaver dam development could aggrade a highly incised stream and improve habitat quantity and quality. Our focus here is to evaluate whether this manipulation resulted in an increase in juvenile steelhead density, growth, survival, and production.

## Watershed-Scale Manipulation

Our experiment was conducted in the lower 32 km of Bridge Creek, a 710 km^2^ watershed draining into the John Day River in north-central Oregon, USA. (Fig. 2). Steelhead are anadromous *Oncorhynchus mykiss* and are the targeted species for recovery in this watershed (hereafter referred to by their freshwater life stages as juveniles or spawners). Prior to the manipulation, steelhead habitat in Bridge Creek exhibited low complexity and poor quality. Most of the mainstem and lower tributary reaches of Bridge Creek were deeply incised, with riparian vegetation limited to a narrow band along the stream^8^. The stream morphology consisted of a plane-bed system with gradients from 0.5–3.0%, very poor pool habitat, and substrate dominated by coarse and embedded gravel and cobble. In addition, stream temperatures in the summer were warm for juveniles, with the lower portion of the study area approaching lethal thermal limits (~26 °C).

Previous research indicated that aggradation behind beaver dams in Bridge Creek can be rapid, and that connection to inset floodplains could be achieved within a decadal scale^8^. However, surveys of beaver dam distributions spanning the last 3 decades showed that dams within Bridge Creek are generally short lived^20^. Due to the lack of large woody riparian vegetation, beaver dams in Bridge Creek were made with small-diameter materials (e.g., willow shoots). Consequently, dams consistently failed (e.g., 1–2 year lifespan) when subject to the typical annual flood in which all the flow energy was concentrated on the dams, as opposed to spreading out over a floodplain.

**Figure 1:**
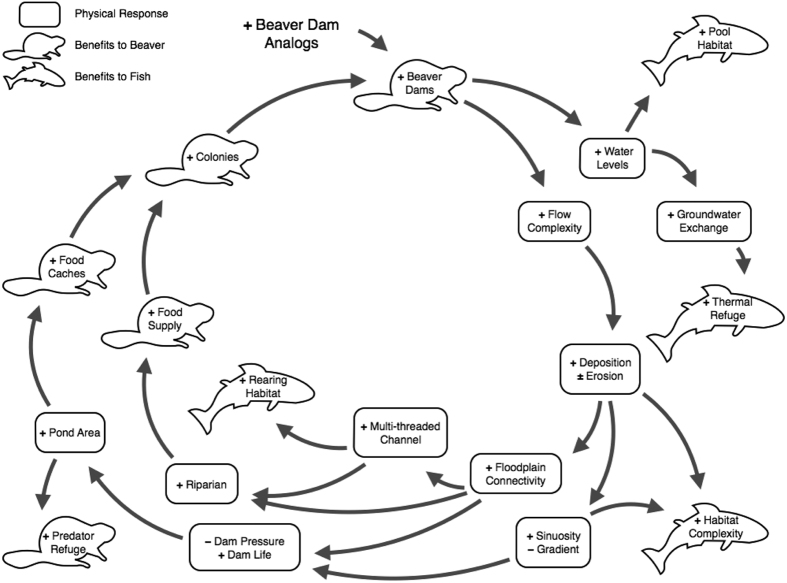
Expected changes following the installation of beaver dam analogs (BDAs). Beaver-made dams and BDAs slow and increase the surface height of water upstream of the dam. Beaver ponds above, and plunge pools below dams change the plane bed channel to a reach of complex geomorphic units providing resting and efficient foraging opportunities for juveniles. Deep pools allow for temperature stratification and greater hydraulic pressures forcing downwellings to displace cooler groundwater to upwell downstream, increasing thermal heterogeneity and refugia. Dams and associated overflow channels produce highly variable hydraulic conditions resulting in a greater diversity of sorted sediment deposits. Gravel bars form near the tail of the pond and just downstream from the scour below the dam, increasing spawning habitat for spawners and concealment substrates for juveniles. Complex depositional and erosional patterns cause an increase in channel aggradation, widening, and sinuosity and a decrease in overall gradient, also increasing habitat complexity. Frequent inundation of inset floodplains creates side channels, high-flow refugia and rearing habitat for young juveniles, and increasing recruitment of riparian vegetation. Flows onto the floodplain during high discharge dissipates stream power, and the likelihood of dam failure. The increase in pond complexes and riparian vegetation increases refugia for beavers, their food supply and caching locations, resulting in higher survival, and more persistent beaver colonies. Beaver will maintain dams and the associated geomorphic and hydraulic processes that create complex fish habitat.

Our goal was to encourage beaver to build on stable structures (i.e., BDAs) that would increase dam life spans to facilitate channel aggradation, and eventually floodplain creation and reconnection^14^. BDAs were built by pounding wooden fence posts vertically into the channel bed and potential floodplain surfaces. Posts were spaced 0.3–0.5 m apart and at a height intended to mimic the crest elevation of an active beaver dam^21^. Willow branches were woven between the posts, and bed sediment was used to plug the base of structures. BDAs were designed to partially replicate many of the basic functions of a natural beaver dam (Fig. 3). The treatment design aimed to saturate four distinct reaches with BDAs, thereby providing resident beavers stable platforms that would encourage the establishment of stable multi-dam complexes to support persistent colonies (Fig. 1). This meant we added BDAs at the maximum frequency that beaver dams are found under natural conditions for a similar stream size and gradient^1^. For most situations at the project site, water from a downstream structure is backed up to the base of the structure upstream during average discharge.

When BDAs were introduced we expected to effectively increase the number and longevity of functional natural and acting beaver dams that, in turn, would initiate a series of alterations that would ultimately restore processes that maintain a new stable state of floodplain reconnection^14^. Changes in both the quantity and quality of fish habitat accompanying this process were expected to elicit a fish population level response (Fig. 1).

**Figure 2:**
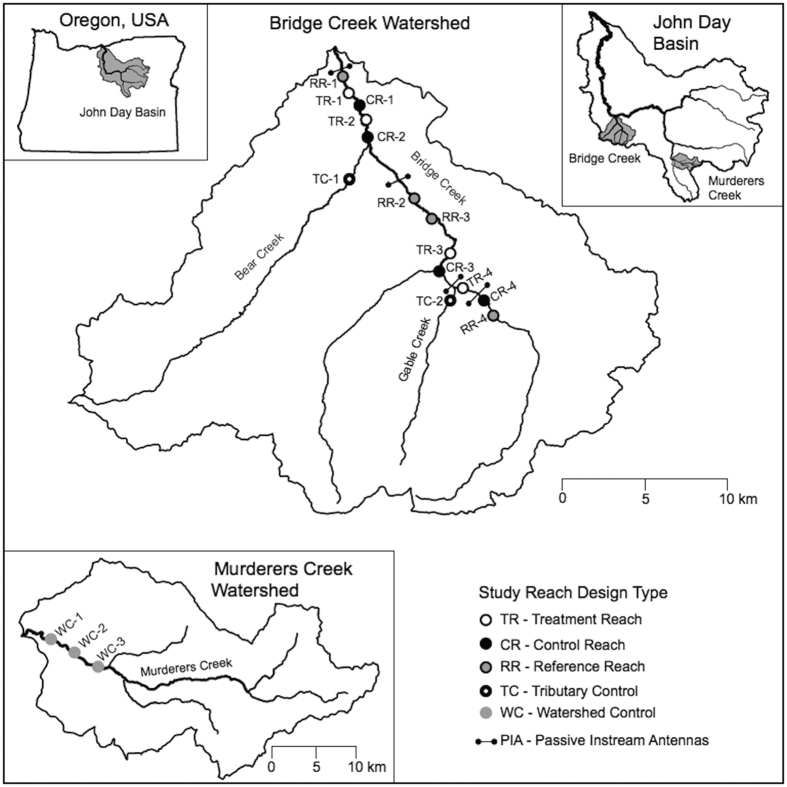
Map of the study areas. TR and CR dots represent treatment and control (similar to treatment reaches with beaver activity) study reach location. RR represent reference study reaches, which generally have minimal inset floodplains and minimal beaver influence. Reaches in tributaries to Bridge Creek (TC) and Murderers Creek (WC) served as additional controls. Passive Instream Antennas (PIAs) distributed throughout Bridge Creek detect Passive Integrated Transponder (PIT) tagged fish to determine viability and movement. Maps were created in ArcGIS version 10.1 (http://desktop.arcgis.com/) and Pixelmator version 3.4 (http://www.pixelmator.com/mac/).

**Figure 3:**
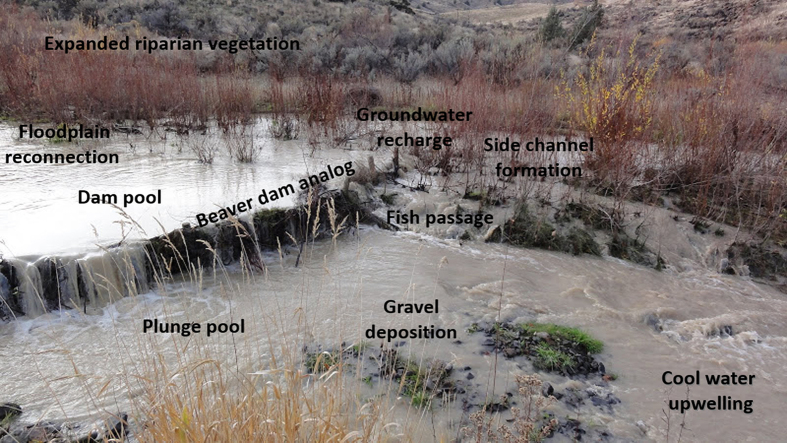
Example of a beaver dam analog (BDA) annotated with some of the expected responses.

The manipulation was implemented in a hierarchical^22^ experimental design where we established four of each treatment, control (both in the early phase 3 stage) and reference (in the early phase 2 stage with minimal beaver influence) reaches within Bridge Creek (Fig. 2). We also selected one control reach in each of two tributaries to Bridge Creek, and three reaches in a control watershed, Murderers Creek (Fig. 2). To assess localized habitat and steelhead responses we made comparisons between treatment, control, and reference reaches within Bridge Creek and its tributaries. To assess population level responses, we compared changes in juveniles in Bridge Creek (across all reach types) to Murderers Creek.

We monitored for three years pre-manipulation (2007–2009) and four years post-manipulation (2010–2013). We conducted an annual census of beaver dams and BDA locations and documented functionality. We monitored fish habitat attributes at sites within reaches once per year. Aerial imagery from 2005 and 2013 was also used to quantify changes in channel area and morphology. We monitored sites for juveniles, which were collected and tagged with Passive Integrated Transponder (PIT) tags each year in June, September, and January. In addition, we compared juvenile densities in impounded and unimpounded portions of three reaches in August and September of 2013 to evaluate their use of these different habitats. We also captured and PIT tagged spawners at a fish weir installed during their upstream migration in lower Bridge Creek (Fig. 2). Recapture of tagged fish provided information on density, growth, survival, and production, as well as the ability of spawners and juveniles to migrate throughout the study area. In general, we used intervention analyses to evaluate changes in habitat and fish responses pre- versus post-manipulation relative to controls^23^ (see Methods for more details).

## Results

### Beaver Dam and BDA Abundance

**Figure 4:**
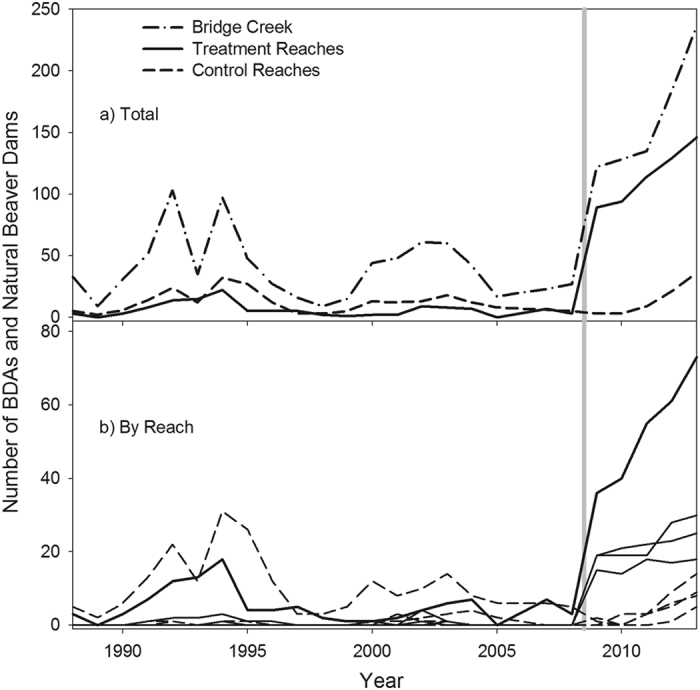
The number of dams (natural beaver dams and BDAs) through time. Upper panel represents the total number of dams for the Bridge Creek (dashed-dotted line), the sum of all treatment (solid line) and all control (dashed line) reaches. The lower panel is total number of dams for each of the four treatment (solid lines) and four control (dashed lines) reaches. Grey vertical line represents when BDAs were initially installed.

Twenty years of beaver dam surveys in the study area prior to 2009 indicates dam-building activity was highly variable (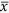 = 40 dams counted per year, min = 9, max = 103, SD = 25; Fig. 4). After 2009, the year in which BDAs were first constructed, the total number of dams (natural beaver dams and BDAs) was on average four times more abundant than pre-manipulation (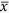 = 160, min = 122, max=236, SD=43; Fig. 4). In 2009, 76 BDAs were installed over 3.4 km of stream in the four treatment reaches. During 2010–2012, additional BDAs were built to replace those that failed during the first year and to continue the stream on the trajectory towards floodplain reconnection (e.g., added on top of BDAs buried by aggradation or to newly formed side channels). By 2012, 121 BDAs were functioning. Of the 236 total dams in Bridge Creek in 2013, nearly half (n = 115) were made by beavers. A total of 171 natural beaver dams and dams built on BDAs represents an 8-fold increase over the 2005–2008 pre-manipulation beaver dam average. The substantial increase in natural beaver dams occurred two years following the manipulation, primarily outside the treatment reaches (Fig. 4), suggesting the manipulation may have created a source of beavers for dispersal into unmanipulated areas. One control reach was subject to a high intensity flood event from an incoming tributary which greatly increased the number of new channels throughout the floodplain and was quickly occupied by beaver. With the exception of this reach, beaver dams in control reaches had a 10-fold higher failure rate than reinforced dams, similar to pre-manipulation conditions. No beaver dams were built in the four reference reaches during the study, however, occasionally dams were found in similarly incised channels elsewhere in Bridge Creek.

### Habitat Response

Following the manipulation, habitat quantity and quality increased in treatment reaches and most control reaches with expanded beaver occupation relative to non-beaver-occupied reference reaches. BDAs and beaver ams both quickly raised the water, and created large upstream dam pools and downstream plunge pools. Relative to our reference reaches and Murderers Creek this resulted in a higher pool frequency (1.04 90% CI ± 1.01 pools/100 m, p = 0.09 and 1.43 90% CI ± 1.51 pools/100 m, p = 0.11, respectively; Supplementary Information Fig. 1) and deeper pools (0.10 90% CI ± 0.054 m, p = 0.02 and 0.162 90% CI ± 0.081 m, p = 0.01; respectively; Supplementary Information Fig. 2). Aggradation occurred rapidly, sometimes burying structures and channels, resulting in newly formed channels. From 2005 to 2013, inundation area of treatment reaches increased by 228%, considerably more than the control and reference reaches which increased 122% and 34%, respectively. New side channels were also formed as high flows were often forced onto inset floodplains. Area of side channels increased in treatment reaches by 1216%, but only by 479% in control reaches, with virtually no change in references reaches.

Information from groundwater wells demonstrated a raising of the water table in a treatment reach relative to a control reach. Water levels below the land surface over the low-flow period averaged −2.527 90% CI ± 0.052 m and −1.909 90% CI ± 0.077 m in a control reach (CR-4) and treatment reach (TR-4), pre-manipulation, and −2.402 90% CI ± 0.121 m and −1.531 90% CI ± 0.169, respectively, post- manipulation. This equates to a 0.25 m (p < 0.001) increase in groundwater levels following the manipulation in our treatment reach relative to our control reach that also had some beaver activity post-manipulation.

Temperature loggers placed at the top and bottom of reaches indicated that temperature either dropped or remained constant as water traversed reaches with extensive beaver dams; whereas, temperatures increased in reaches without beaver dams. Maximum temperatures were on average 1.47 °C (90% CI 1.34 to 1.72, p < 0.001) cooler in reaches that gained beaver dams after the manipulation (0 dams pre-manipulation to an average of 6.7 dams within 500 m upstream of the temperature loggers post-manipulation), than a reference reach that had no beaver dams within 500 m upstream over the study period.

**Figure 5:**
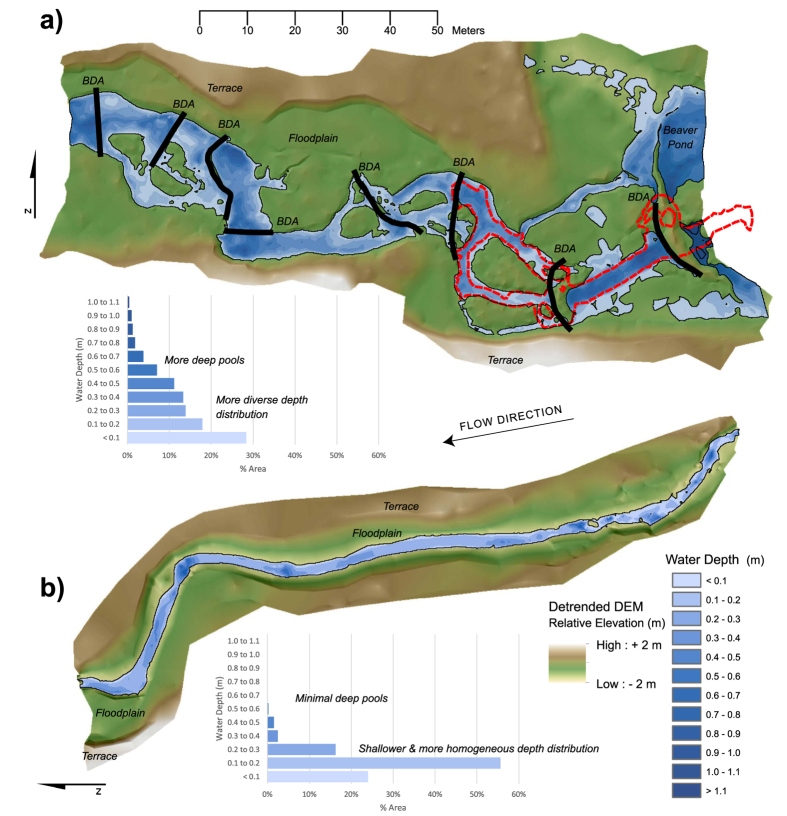
Water depth maps, relative topography and depth distributions for habitat sample site in treatment reach TR-4 (**a**) and a reference reach RR-4 (**b**). Digital elevation models (DEMs) were built from data collected from 2013 topographic surveys, with bottom elevations subtracted from water surface elevations to obtain water depths. Red outline in a) is the location of temperature survey information depicted in Fig. 6. Figure was created in ArcGIS 10.3 and Adobe Illustrator CS6.

**Figure 6:**
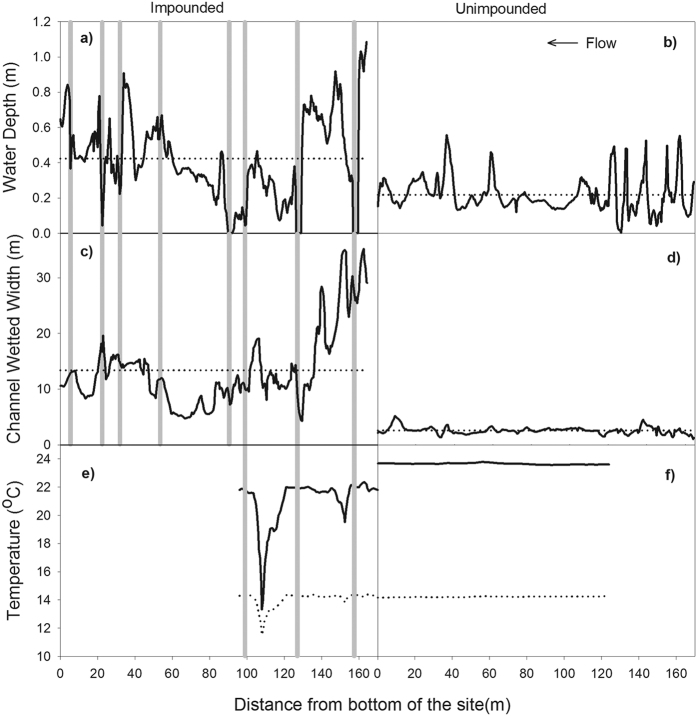
Longitudinal profile of stream characteristics. Water depth and channel width was determined from topographic survey information in 2013 in impounded TR-4 (panel a & c) and unimpounded RR-4 (panel b & d) sites, solid line is the metric value for each location, dotted line is the mean value for the reach. Longitudinal temperature profiles (panel e & f) were obtained from multiple temperature loggers in TR-4 (see Fig. 5) and an unimpounded reach just upstream (between TR-4 and CR-4). The solid line is maximum and dotted line is minimum temperatures. Grey vertical lines represent the locations of dams.

For illustrative purposes regarding changes in channel planform, we compare water depth maps and longitudinal profiles of sites within the treatment reach (TR-4) and the closest upstream surveyed non-beaver-occupied reference reach (RR-4). Water depth maps and distributions depict greater variability in water depths, channel complexity, and an increase in the number of side channels in the treatment site (Fig. 5). Longitudinal profiles also emphasize differences in the variability of channel width and depths (Fig. 6a–d). We also compared day and night longitudinal temperature profiles for a site in TR-4 to a non-beaver-occupied site approximately 0.5 km upstream. During both day and night, the treatment site was cooler and contained considerably greater thermal heterogeneity (including cool refugia) than the unimpounded site which exhibited almost no longitudinal variability (Fig. 6e,f).

### Fish Population Response

**Figure 7:**
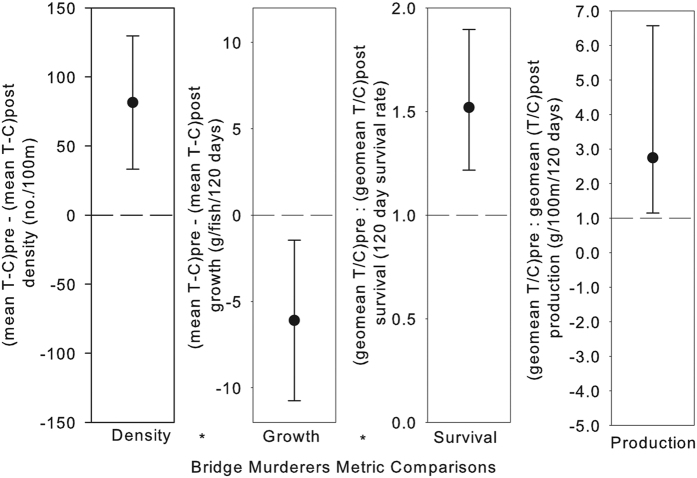
Summary of intervention analyses for juvenile steelhead responses. On every sampling occasion, the control (C) is subtracted (difference) or divided into (ratio) the treatment (T) value. Next, the average difference pre-manipulation is subtracted (difference) or divided into (ratio) the post-manipulation value. Confidence intervals (90%) not overlapping zero for difference and 1 for ratio indicates significance at α  = 0.1. Comparisons are made between Bridge Creek (treatment) and Murderers Creek (control), respectively. Results for difference in density and average growth, and ratio of survival and production (estimated as density*growth*survival) are displayed.

We PIT tagged 35,867 juveniles from 2007 to 2013. When comparing a beaver pond to an adjacent upstream free-flowing site in three reaches on two dates, the linear and areal density of juveniles was on average 210 fish/100 m (p = 0.007) and 27 fish/100 m^2^ (p = 0.004) greater in impounded than unimpounded reaches, suggesting a higher preference by juveniles for ponded areas. After the manipulation, fish density increased in Bridge Creek by 81 fish/100 m relative to our control watershed of Murderers Creek (p = 0.01; Fig. 7 and Supplementary Information Figs 3 and 4). In contrast, juvenile growth decreased after the manipulation by 6.1 grams per season in Bridge Creek relative to Murderers Creek (p = 0.036; Fig. 7 and Supplementary Information Fig. 5). Both Bridge and Murderers Creek exhibited density-dependent decreases in growth (growth = −0.001*density + 0.215, R^2^ = 0.59, p < 0.0001; growth = −0.001*density + 0.188, R^2^ = 0.27, p = 0.02, respectively). Following the manipulation, juvenile survival increased by 52% in Bridge Creek relative to Murderers Creek (p = 0.004; Fig. 7 and Supplementary Information Fig. 6). Production of juveniles, being the product of density, growth, and survival, is an informative quantitative indicator of population performance because it integrates multiple responses^24^. Just four years after the manipulation, there was an increase of 175% in juvenile production in Bridge Creek, relative to Murderers Creek (p = 0.06; Fig. 7 and Supplementary Information Fig. 7).

Despite the dramatic increase in beaver dams and BDAs, we observed no changes in upstream spawner migration success based on detections of PIT-tagged spawners at upstream arrays. Prior to the manipulation 57%, 18%, and 17% (92% total) of tagged spawners were detected above PIAs 2 through 4, respectively (the spawner trap is located at PIA1). After the manipulation, we observed, on average, 49%, 31%, 14% (93.5% total) of the tagged spawners above these detection sites. Furthermore, several spawners were documented as having passed more than 200 dams and BDAs during their migrations. Likewise, more than 1000 PIT-tagged juveniles migrated downstream past the lower-most PIT tag array (PIA1) each year, the near expected amount given observed survival estimates and antenna efficiency. While upstream movement of juveniles is not common in Bridge Creek, we re-detected individuals in upstream reaches separated by more than 40 dams. Overall, mark-resight data indicate that neither beaver dams, nor BDAs, are barriers to spawner or juvenile movement.

## Discussion

The addition of BDAs into Bridge Creek led to an immediate and rapid increase in the number of natural beaver dams, not only in our treatment areas but throughout much of Bridge Creek. Beavers build dams and dig canals to expand deep water to create refugia and to aid in the transport of the woody vegetation they harvest. We believe this increased activity throughout Bridge Creek was, in part, due to an increase in the population of beavers facilitated by BDAs. These structures provided stable places to build and expand natural beaver dam complexes that improved their habitat. Changes in the abundance of beavers are difficult to quantify because of their ability to quickly learn to avoid traps^25^. Thus, we cannot state with certainty that the beaver population actually increased following the installation of BDAs. Whether their dam-building activities increased because of a demographic or behavioral response is somewhat immaterial, because the modification of the stream ecosystem, rather than the beavers themselves, likely caused the fish population response.

BDAs and beaver dams led to large changes in both fish and beaver habitat, and the steelhead population response largely followed our hypothesized pathways (Fig. 1). We found compelling evidence that beavers increased the quantity of juvenile habitat. We observed higher linear and areal densities of juveniles in impounded sections of stream relative to unimpounded sections. To demonstrate the potential for beavers to alter stream salmonid production, we believe linear density is the most indicative numeric response variable because dams increase the area of fish habitat per length of stream. Areal densities normalize across streams of different widths; thus a fish response might not be detected even if the population increased simply by increasing the width of the same length of stream (i.e. areal densities stayed the same or even decreased). Studies reporting the influence of beaver ponds to produce more fish relative to other habitat types often use areal densities^26^,^27^. An areal density response metric may under-represent the contribution this habitat type has to the population, because one mechanism by which beaver dams increase fish abundance is by increasing the quantity of fish habitat, as we observed.

Natural beaver dams and BDAs increased the area of juvenile habitat in the treatment reaches in Bridge Creek because these reaches were in the building of the inset floodplain phase (early phase 3) of the successional cycle of an incised channel. The combination of increasing the dam crest height up to the inset floodplain and channel aggradation behind the dam, allowed surface waters to spill out onto inset floodplains greatly increasing the habitat area. The benefits of creating more fish habitat would be diminished in an incised trench, because small increases in surface water area occurs as surface water elevation increases. This condition is representative of our reference reaches. However, beaver dams and BDAs likely increase the rate at which phase 2, or channel widening occurs, thus accelerating the channel incision recovery process to benefit fish populations^14^. In fact, we most commonly observe breaches on the ends of beaver dams or BDAs. Such breaches create an acceleration of a flow jet at the outside bank of the incision trench and increases the rate of widening and the sinuosity of the channel.

The increase in groundwater elevation surrounding beaver ponds likely results in increased flow throughout the summer as water is slowly released^28^,^29^. We also found that water temperatures stayed the same or decreased throughout reaches with beaver ponds, and that diel fluctuation was dampened. Because dams slow water and often increase the area of solar input, a common assumption is that temperatures increase in impounded reaches^30^. However, quantitative evidence supporting^31^,^32^ or refuting^33^ this claim suggests that the complex interaction of solar input, and exchange with the hyporheic or groundwater call into question this simple generality^29^. In Bridge Creek, increased residence time and the slowed release of potentially cooler water after the construction of BDAs also increases habitat quantity during times of very low discharge observed during hot summer conditions.

Increasing habitat complexity may also partially explain the observed increase in total juvenile abundance, survival and productivity. In sections with natural and simulated beaver dams, we observed higher variability in water depth, channel width, and temperature from dam-building activities, all indicators of increased habitat complexity. Increased habitat complexity provides fish a greater selection of locations at which to forage, rest, and avoid predation and high flow events, while reducing migration distances required to conduct these activities for multiple life-stages^34^. Thus, we suspect that an increase in habitat complexity is partly responsible for the observed positive steelhead population responses.

This study provides further quantitative support to the proposal to reintroduce or expand beaver populations in their native range in North America and Eurasia to recover incised channels^8^,^14^,^35^. However, the impacts of beaver reintroductions on fish populations, summarized in a recent review^30^, have been debated. Of note is the paucity of rigorous empirical studies backing conclusions of both positive and negative impacts. Unfortunately, many approaches to managing beaver populations for fisheries enhancement are also based on assumptions or results from weak study designs. In fact, policies to remove beavers/beaver dams as a means to improve salmonid populations, still exist in some U.S. states^36^. This does beg the question, how did both beavers and salmonids coexist in far greater numbers than occurs today without human intervention? While we observed many of the commonly reported positive impacts (habitat complexity), many of the claims of negative impacts of beaver dams on fish (e.g., fish passage barriers, temperature increases) are not supported by our findings to date.

The factors contributing to variability in fish and habitat responses across systems deserves further inquiry and will only be illuminated as additional studies are pursued in widely varying systems. For example, one large scale study found evidence suggestive of an increase in brook trout production after the removal of 200 beaver dams maintained for over two decades, in a low gradient stream network in Wisconsin, USA.^37^. In low-gradient systems with a reduced range of water velocities, beaver dams may not create the same heterogeneous environment as they do in relatively higher gradient systems like Bridge Creek. Multiple controlled experimental manipulations or comparative studies across a range of stream gradients would help establish whether salmonid and fish community responses to beaver-dominated systems are gradient dependent.

The use of BDAs to provide or enhance the benefits beavers have on stream ecosystems and salmonids could be a potential restoration strategy but requires additional rigorous assessments elsewhere. The use of BDAs as a restoration approach is certainly attractive from a cost perspective^38^. In a stream like Bridge Creek, installation of a BDA takes three people approximately 1–4 hours to install, requires a hydraulic post driver and 20–40 wood posts, (at ca. US$4 per post). The cost at a density of ~30 BDAs per km is less than $11,000. In contrast, conventional restoration techniques to achieve such objectives often involve massive grading operations with heavy equipment and major revegetation efforts that are extremely expensive and uncertain. Not only was our manipulation large in scale, but we benefited from the help of beaver to maintain, and likely improve, structures until self-maintaining processes (e.g. floodplain connection) were restored.

More important than the feasibility is our demonstration that such a restoration strategy actually results in benefits to the target population. Billions of dollars are spent annually on stream restoration in the U.S. alone^39^; however, very few studies have documented changes beyond localized increases in fish abundance following stream restoration^40^. Far fewer demonstrate increases in responses associated with fitness (i.e., survival, growth, and production). The few studies that have detected positive population-level changes due to restoration were likely able to do so because they were conducted at large spatial and temporal scales (many km and 10+ years), included extensive monitoring, and maximized contrasts (e.g., before-after-control-intervention experimental designs)^41^,^42^. Our ability to detect a fish response was, in part, due to the large signal created by adding BDAs to nearly 4 km of Bridge Creek, coupled with considerable localized changes caused by both BDAs and natural beaver dams. Although we tagged >35,000 juveniles, reach-level comparisons were difficult to make for responses requiring seasonal recaptures such as survival, growth, and production. We believe that large-scale experimental manipulations, rather than reach-level, opportunistic evaluations of small-scale habitat projects are necessary to increase our understanding of how fish respond to changes in their habitat or provide evidence of restoration benefits.

In order to improve our understanding of how organisms respond to their environment, ecosystem experiments that use restoration as a treatment and incorporate appropriate large-scale controls should be actively pursued. This approach is consistent with experimental and adaptive management and has recently been implemented to test the effects of stream restoration in several watersheds^19^. Effective implementation of this experimental restoration approach requires an investment in coordination, strong experimental designs, cost-effective yet extensive restoration strategies, and directed monitoring and research. However, the potential to implement more effective management and restoration actions while learning from such approaches readily justifies their cost.

## Methods

### Experimental and Survey Design

The manipulation was implemented in a hierarchical^22^ experimental design where we compared four treatment and four control reaches in the early phase 3 stage within Bridge Creek (Fig. 2). We identified four additional reference reaches with minimal beaver influence. To address effects at different scales, issues of potential non-independence, and to protect against loss of control site information (i.e., create redundancy), we selected one control reach in each of two tributaries to Bridge Creek, and three reaches in a control watershed, Murderers Creek (Fig. 2). All experimental reaches were between 500 and 2000 m in stream length.

We monitored for three years pre-manipulation (2007–2009) and four years post-manipulation (2010–2013). Sample sites (i.e. segments within reaches) were used to characterize reaches. We monitored sites once a year for fish habitat. Aerial imagery from 2005 and 2013 was also used to quantify changes in channel morphology. We monitored sites for juveniles, which were collected and tagged with 12 mm full duplex Passive Integrated Transponder (PIT) tags each year in June, September and January. A habitat preference study to compare densities of juveniles in impounded and unimpounded portions of three reaches was conducted in the fall of 2013. We captured spawners during their upstream migration at a fish weir located near the mouth of Bridge Creek (Fig. 2). All fish PIT tagged were weighed and measured, and spawner sex was determined. Recapture of tagged fish provided information on movement, density, growth, and survival. We estimated production as the product of these responses. In general, we used intervention analyses to evaluate changes in fish response following the manipulation relative to controls^23^.

### Beaver Dam Surveys

Beaver dam census surveys were enumerated throughout the study area on Bridge Creek in late December during each year from 1988 to 2013^20^. During these surveys, beaver dams were recorded as being either intact (actively impounding water in pond to the maximum dam crest elevation), breached (partially impounding water) or blown out (not impounding water). When BDA structures were installed in 2009 they were surveyed in the same manner as natural beaver dams, and whether or not BDAs were being actively maintained by beavers was also recorded. These surveys were used to track the abundance and distribution of natural dams and BDA structures being maintained by beaver throughout the control, treatment, and reference reaches of Bridge and Murderers Creek (Fig. 4).

### Habitat Surveys

Fish habitat surveys were conducted in November of each year at a single site within each of the reach types, as well as on rotating basis (every other year) at supplementary sites. In total 48 sites within Bridge Creek and 3 sites in Murderers Creek were sampled. Sites were 160 m in length (approximately 20 bankfull widths) and were surveyed using the methods developed by the Columbia Habitat Monitoring Program^43^. These surveys quantify a number of fish habitat attributes, and utilize survey-grade equipment to provide channel and floodplain topography and water surface extent and elevation. Topographic data were used to generate 10 cm resolution Digital Elevation Models (DEMs) of channel and water surface elevations that were differenced to create a third surface representing the water depths throughout each sub-site survey (Fig. 5). Longitudinal profiles of water depths and channel widths were extracted from water depth maps and wetted widths calculated at an interval of 0.5 m along the channel thalweg from the bottom to the top of the site (Fig. 6a–d).

Channel inundation area was calculated from high-resolution (15 cm) aerial imagery of Bridge Creek before and after the manipulation occurred and beaver dams proliferated. Aerial imagery was acquired on September 27, 2005 and a repeat acquisition was conducted on May 5, 2013 (Watershed Sciences, Corvallis, Oregon). Following acquisition, imagery was ortho-rectified and subject to rigorous quality assurance procedures to ensure spatial accuracy. Areas of inundation were extracted from the 2005 and 2013 aerial imagery by digitizing the extent of the wetted channel throughout each study site using ArcGIS.

Temperature loggers (Onset Tidbit V2, U22) were deployed at the top and bottom of all reaches, continuously recording temperature every 15 minutes. In addition, longitudinal stream temperature profiles were created from temperature monitoring in a portion of a site in a treatment and reference reach (Fig. 6e,f). Temperature loggers were fixed to the streambed for two weeks during the summer throughout the wetted channel at a density of approximately 0.04 m^2^, and the location of each logger was surveyed using a Real Time Kinematic (RTK) GPS. Temperature information from each logger was used to construct digital temperature models depicting the spatial distribution of daily maximum and minimum temperature throughout the reach. The longitudinal profiles of stream temperatures presented in Fig. 6e,f, were created by extracting the maximum and minimum temperature on August 17, 2012 observed along the channel thalweg at an interval of 0.5 m from the bottom to the top of the surveyed reach.

Well fields were established adjacent to reaches TR-4 and CR-4 to compare groundwater elevational changes pre- and post-manipulation between a treatment and control reach. A line of 2 to 3 wells perpendicular to the channel extended back approximately 70 m on the terrace. Four and three lines of wells (lines were spaced 50–70 m apart parallel to the stream) produced 10 and 9 wells for the treatment and control reach, respectively. Groundwater elevation was obtained from wells drilled approximately 12 m deep and lined with 5 cm slotted PVC. In each well, water table elevation and groundwater temperature data were collected using HOBO Water Level Loggers (Onset Computer Corp., model U20-001-01) set to record data in one or two hour intervals over the duration of the study period.

### Seasonal Juvenile Steelhead Surveys

Juvenile steelhead surveys were conducted in all reach types. Survey sites within these reaches ranged between 500–1000 m in stream length. On each juvenile steelhead survey occasion, two electrofishing passes were conducted, separated by a 24-hour period. During each pass juvenile steelhead were captured using a backpack electrofisher (SAMUS-725MP) and dip nets while fishing from the bottom to the top of the site. Captured salmonids ≥70 mm were anesthetized, measured (mm), weighed (g), and PIT tagged (Biomark HPT12, Boise, Idaho) in the abdominal cavity, then released back to their approximate capture location following recovery from the anesthetic. Methods of fish capture and handling were approved by the National Oceanic and Atmospheric Administration’s Biological Opinion in accordance to their Federal Columbia River Power System Biological Opinion Letters of Determination 22-14-NWFSC100 and 23-14-NWFSC101 Scientific Research Permits.

Recapture information from each of the two electrofishing passes was used to estimate the population size of juvenile steelhead residing in each site during each seasonal sampling occasion using the Chapman equation^44^. In some cases, low steelhead densities prevented recapture of tagged individuals, and an estimate of capture efficiency (no. marked fish/no. of recaptures) calculated for each site from previous sampling occasions was used to expand the number of fish captured during the first pass into an estimate of population size.

Although the Cormack-Jolly-Seber (CJS) model has traditionally been used to estimate survival rates for tagged fish in the Columbia River Basin, it does not account for emigration thus producing estimates of apparent rather than true survival. Additionally, CJS cannot accommodate continuously collected data, such as the resightings from passive instream antenna (PIAs) that constitute a large portion of our resight data. Therefore, we used the Barker model^45^ that uses recapture and continuous “resight” information to simultaneously estimate rates of emigration, immigration, and survival to produce estimates of true survival^46^.

We generated encounter histories for each individual PIT-tagged fish from active tagging, mobile antenna surveys, and continuous detections from PIA arrays. We used Akaike’s Information Criterion corrected for small sample size (AICc)^47^,^48^ to determine the most parsimonious model for recapture/resight and movement parameters in the Barker model, while survival parameters were unconstrained (i.e., varied through time) in all models. Survival estimates and 95% credible intervals were computed using the Markov Chain Monte Carlo (MCMC) procedure in Program MARK^48^,^49^. Seasonal survival rates were standardized to 120 days.

Juvenile steelhead growth rates were calculated by direct measurement of the change in weight of PIT-tagged individuals recaptured from one season to the next (reported as g/fish/120 d). Seasonal production (g/100 m/120 d) of juvenile steelhead was calculated for each site as the product of the beginning of season density, seasonal growth rate, and seasonal survival.

### Analyses

We evaluated differences in pool frequency, residual pool depth, temperature, and groundwater elevation, as well as fish responses between treatments and controls using Before-After-Control-Impact paired (BACIP) design intervention analyses^50^. These comparisons were made at the reach or watershed scale depending on the response. Controls in this sense are used as covariates where effects common to both treatment and control reaches (e.g. weather) are filtered from the treatment time series of information by subtracting the control value from the treatment value for all observations. The average of this difference pre-manipulation is compared to the average of the value post-manipulation using a t-test. An α = 0.10 was used to create 90% confidence intervals. Intervals encompassing zero were taken to indicate a lack of significant pre- versus post-manipulation difference for each response variable (Fig. 7 and Supplementary Information Fig. 3). In the case of survival and production, a natural log transformation was necessary to meet assumptions of normality (evaluated by inspecting quantile to quantile plots of residuals), which is equivalent to using treatment:control ratios for each observation event in the time series and conducting a ratio t-test. If the 90% confidence intervals surrounding the ratio crosses 1 then a significant difference was not observed.

These types of intervention analyses can bias p-values if assumptions of additivity and serial independence are violated^50^,^51^. To test the assumption of additivity, the presence of trends between the average versus the difference in paired treatment-control observations was evaluated for each response^50^. To test for auto-correlation, the difference between a treatment-control pair at time *t* was compared to the difference at *t* + 1, for all observations^50^. A significant positive correlation between *t* and *t* + 1 observations was taken as evidence for auto-correlation, suggesting that our p-values were negatively biased. In this case, we also noted whether a positive temporal trend in the difference between treatment-control pairs during the before period, as this violation of the additivity assumption is particularly egregious^52^.

## Additional Information

**How to cite this article**: Bouwes, N. *et al*. Ecosystem experiment reveals benefits of natural and simulated beaver dams to a threatened population of steelhead (*Oncorhynchus mykiss*). *Sci. Rep.* **6**, 28581; doi: 10.1038/srep28581 (2016).

## Supplementary Material

Supplementary Information
