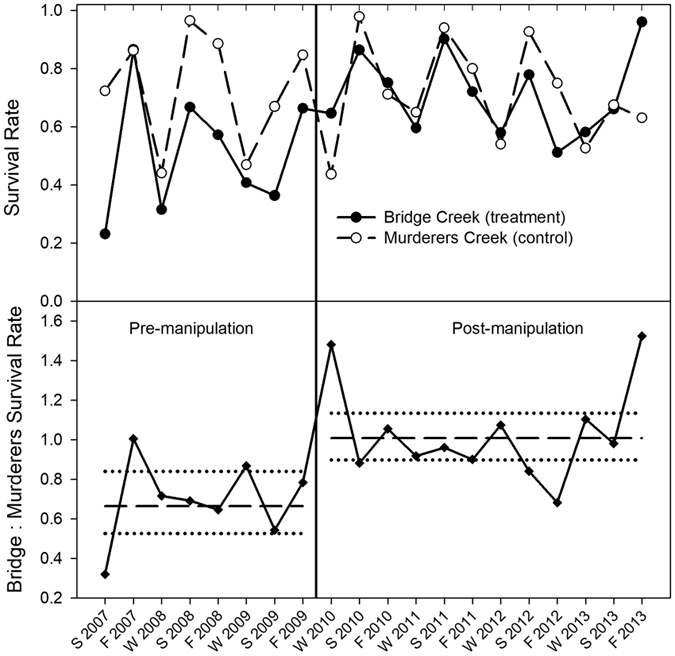# Corrigendum: Ecosystem experiment reveals benefits of natural and simulated beaver dams to a threatened population of steelhead (*Oncorhynchus mykiss*)

**DOI:** 10.1038/srep46995

**Published:** 2018-06-05

**Authors:** Nicolaas Bouwes, Nicholas Weber, Chris E. Jordan, W. Carl Saunders, Ian A. Tattam, Carol Volk, Joseph M. Wheaton, Michael M. Pollock

Scientific Reports 6: Article number: 2858110.1038/srep28581; published online: 07 04 2016; updated: 06 05 2018

This Article contains an error, where Supplementary Information Figure 6 is a duplicate of Supplementary Information Figure 7. The correct Supplementary Information Figure 6 appears below as Fig. 1.

## Figures and Tables

**Figure 1:**