# Supplementary material for: Ecosystem experiment reveals benefits of natural and simulated beaver dams to a threatened population of steelhead (Oncorhynchus mykiss)
Source: Sci Rep. 2016 Jul 4;6:28581. doi: 10.1038/srep28581 (PMC4931505; doi:10.1038/srep28581)
Supplement: Supplementary Information [file srep28581-s1.doc]

**Supplementary Information**

**Ecosystem experiment reveals benefits of natural and simulated beaver dams to a threatened population of steelhead (*Oncorhynchus mykiss*)**

Nicolaas Bouwes^1,4*^, Nicholas Weber^1^, Chris E. Jordan^2^, Michael M. Pollock^3^, W. Carl Saunders^1,4^, Ian A. Tattam­^5^, Carol Volk^6^, and Joseph M. Wheaton^4^

**Pool habitat responses**

After the addition of beaver dams and BDAs, plunge pools were formed downstream of the dam. Dam pools also formed as dams ponded water upstream. Relative to our reference reaches and Murderers Creek this resulted in a higher pool frequency (1.04 ± 1.01 pools/100m, p=0.093 and 1.43 ± 1.51 pools/100m, p=0.11, respectively; Supplementary Information Fig. 1) and deeper pools (0.10 ± 0.054 m, p=0.02 and 0.162 ± 0.081 m, p=0.01; respectively; Supplementary Information Fig. 2). While an increase in 1-2 pools/100m does not represent a substantial increase, the added pools are extremely large, being up to 50m in length.

**Juvenile steelhead responses**

In Bridge Creek, densities of fish were generally much lower in the warm lower sections of the creek (
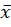
=47.7 fish/100m over all reaches and sampling occasions), compared to the cooler upper sections (
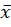
=134.7 fish/100m). In fact, 25% of the 70 abundance observations in the lower four reaches had <3.2 fish/100m, resulting in high relative error in abundance estimates. Therefore, comparisons between treatment and control in the lower reaches exhibited little difference in fish densities (Supplementary Information Fig. 3). Abundance estimates in the upper section of Bridge Creek were based on far more tagged and recaptured fish. In the upper section, treatment reaches exhibited higher fish densities following the manipulation than did the control reaches (Supplementary Information Fig. 3). Fish densities averaged across all treatment control and reference sites in Bridge Creek increased post-manipulation relative to average densities in Murderers Creek (Supplementary Information Fig. 3&4).

Physical recapture of enough fish to provide reasonable estimates of growth were feasible at only the watershed scale, using pooled mark-recapture information from all Bridge Creek sites (median total of 38 juveniles/season recaptured in Bridge Creek) to all Murderers Creek sites (Supplementary Information Fig. 5). Like growth, survival estimates required pooling across the watershed to have sufficient sample size to make comparisons between treatments and controls (Supplementary Information Fig. 6). And finally, because production is the product of all three density, growth and survival, this response also could only be compared by pooling across the watershed (Supplementary Information Fig. 7).

Evaluation of the assumptions of intervention analyses suggested that responses were additive for density, growth, and survival (r^2^=0.02, 0.13, 0.034). A weak, but significant, negative relationship between treatment-control differences to treatment-control averages (r^2^=0.315) was observed for production. We did not observe a trend in the differences in the before period. Non-additivity can result in low power or inflated Type 1 errors. The negative relationship of increased differences in production at low production levels is likely a result of compounding errors in this derived response (product of density, growth, and survival) when low numbers of fish are encountered. If true, this would suggest our p-value is over-predicted. Production does follow the same pattern as the change in both the habitat response, and the density and survival responses, which provides evidence that we observed a biologically meaningful production response that occurred following the manipulation. Auto-correlation was not observed for growth, survival and production (r=0.095, 0.035, 0.17, respectively). Auto-correlation was only weakly observed in the density response (r=0.42). However, density exhibited a p-value of <0.01. Because our observed response was far lower than α=0.1 (our *a priori* acceptable significance level commonly used in field experiments to balance type I and type II errors), we believe a strong density response to the manipulation was observed even with marginal autocorrelation present in the data.

**Supplementary Information** **Figure 1**. Pool frequency (pools/100m) pre- and post-manipulation differences between treatment reaches and reference reaches (top panel) and Murderers Creek (bottom panel).

**Supplementary Information** **Figure 2.** Residual Pool Depth pre- and post-manipulation differences between treatment reaches and reference reaches (top panel) and Murderers Creek (bottom panel).

**Supplementary Information** **Figure 3:** Summary of intervention analyses. On every sampling occasion, control density is subtracted from the treatment density. Next, the average difference pre-manipulation is subtracted from the post-manipulation value. A difference of zero indicates no change (90% error bars not overlapping zero indicates significance at α = 0.1). Comparisons are made between treatment/control pairs within lower Bridge Creek, within upper Bridge Creek, and between Bridge Creek (BC: treatment) and Murderers Creek (MC: control), respectively (see Fig. 2 for reach locations).

**Supplementary Information** **Figure 4:** Time series of juvenile steelhead density (number/100m) estimates for Bridge Creek (treatment) and Murderers Creek (control) watersheds (upper panel) and difference between Bridge Creek and Murderers Creek densities (lower panel) for spring, fall, winter (S,F,W). Vertical line represents the date of the manipulation. In the lower pane, the dashed lines represent the mean value, and the dotted lines the upper and lower 90% confidence intervals, for pre- and post-manipulation.

**Supplementary Information** **Figure 5:** Time series of juvenile average steelhead growth (g/fish/120 days) estimates for Bridge Creek (treatment) and Murderers Creek (control) watersheds (upper panel) and difference between Bridge Creek and Murderers Creek growth (lower panel) for the seasons following spring, fall, winter (S,F,W) sample events. Vertical line represents the date of the manipulation. In the lower pane, the dashed lines represent the mean value, and the dotted lines the upper and lower 90% confidence intervals, for pre- and post-manipulation.

**Supplementary Information** **Figure 6:** Time series of juvenile steelhead survival estimates (proportion surviving after 120 days) for Bridge Creek (treatment) and Murderers Creek (control) watersheds (upper panel) and ratio of Bridge Creek and Murderers Creek survival estimates (lower panel) for the seasons following spring, fall, winter (S,F,W) sample events. Vertical line represents the date of the manipulation. In the lower pane, the dashed lines represent the mean value, and the dotted lines the upper and lower 90% confidence intervals, for pre- and post-manipulation.

**Supplementary Information** **Figure 7**: Time series of juvenile production (g/100m/120 days) estimated as density*growth*survival for Bridge Creek (treatment) and Murderers Creek (control) (upper panel) and ratio of Bridge Creek and Murderers Creek survival estimates (lower panel) for the seasons following spring, fall, winter (S,F,W) sample events. Vertical line represents the date of the manipulation. In the lower pane, the dashed lines represent the mean value, and the dotted lines the upper and lower 90% confidence intervals, for pre- and post-manipulation.
